# Serum nitrated nucleosome levels in patients with systemic lupus erythematosus: a retrospective longitudinal cohort study

**DOI:** 10.1186/ar4477

**Published:** 2014-02-07

**Authors:** Sara Croca, Paul Bassett, Charis Pericleous, Karim Fouad Alber, David Latchman, David Isenberg, Ian Giles, Anisur Rahman, Yiannis Ioannou

**Affiliations:** 1 Centre for Rheumatology Research, Division of Medicine, University College London (UCL), 4th Floor, Rayne Institute, 5 University Street, London, WC1E 6JF, UK; 2 Joint Research Office, UCL/University College London Hospital (UCLH)/Royal Free Hospital London, London, NW3 2QG, UK; 3 Birkbeck, University of London, Malet Street, London, WC1E 6JF, UK; 4 Arthritis Research UK Centre for Adolescent Rheumatology (UCL/UCLH/Great Ormond Street Hospital), London, WC1E 6JF, UK

## Abstract

**Introduction:**

Circulating nucleosomes released from apoptotic cells are important in the pathogenesis of systemic lupus erythematosus (SLE). Both nucleosomes and anti-nucleosome antibodies are deposited in inflamed tissues in patients with SLE. Active inflammation promotes nitration of tyrosine residues on serum proteins. Our hypothesis was that levels of nitrated nucleosomes would be elevated in patients with SLE and could be associated with disease activity. We therefore carried out a retrospective longitudinal study to investigate factors affecting levels of nitrated nucleosomes (NN) in patients with SLE.

**Methods:**

A novel serum ELISA was developed to measure serum NN and modified to measure serum nitrated albumin (NA). Levels of both NN and NA were measured in 397 samples from 49 patients with SLE followed through periods of disease flare and remission for a mean of 89 months. Anti-nucleosome antibody (anti-nuc) levels were measured in the same samples. The effects of 24 different clinical, demographic and serological variables on NN, NA and anti-nuc levels were assessed by univariable and multivariable analysis.

**Results:**

Patients with SLE had higher mean NN than healthy controls or patients with other autoimmune rheumatic diseases (*P* =0.01). Serum samples from 18 out of 49 (36.7%) of SLE patients were never positive for NN. This group of 18 patients was characterized by lower anti-double stranded DNA antibodies (anti-dsDNA), disease activity and use of immunosuppressants. In the remaining 63.3%, NN levels were variable. High NN was significantly associated with anti-Sm antibodies, vasculitis, immunosuppressants, hydroxychloroquine and age at diagnosis. NN levels were raised in neuropsychiatric flares. NN levels did not completely parallel NA results, thus providing additional information over measuring nitration status alone. NN levels were not associated with anti-nuc levels.

**Conclusions:**

NN are raised in a subset of patients with SLE, particularly those who are anti-Sm positive. Elevated NN may be a marker of vascular activation and neuropsychiatric flares in these patients.

## Introduction

Nitration and nucleosomes are both relevant to the pathogenesis of systemic lupus erythematosus (SLE). Nitration may be linked to development of cardiovascular disease (CVD). Patients with SLE have an increased risk of developing CVD [1] for reasons that are not fully understood [2,3]. Nitric oxide (NO) produced by the vascular endothelium is an important metabolite involved in processes such as vasodilatation and inhibition of platelet aggregation [4]. When produced in excess, for example under conditions of systemic inflammation, NO can cause chemical alteration of lipids and proteins. In particular, tyrosine residues within proteins can be nitrated irreversibly, forming nitrotyrosine. In patients with SLE, the serum nitrite level (an index for NO production) correlates with disease activity and levels of anti-double stranded DNA (anti-dsDNA) antibodies [4]. Patients with active lupus nephritis have higher levels of nitrotyrosine than those without renal disease [5,6]. In theory, any serum protein containing tyrosine residues may be nitrated. We are particularly interested in nitration of histones within nucleosomes. Nucleosomes are released during apoptosis and this apoptotic debris is not cleared efficiently in patients with SLE [7]. Both nucleosome and anti-nucleosome antibody levels are elevated in these patients [8] and deposition of nucleosome-anti-nucleosome complexes is important in lupus nephritis [9]. Thus, our hypothesis is that levels of nitrated nucleosomes (NN) in the serum of patients with SLE could rise, particularly during flares of disease activity. We developed a novel enzyme-linked immunosorbent assay (ELISA) to test this hypothesis.

The principle of this novel ELISA is that serum proteins containing nitrotyrosine residues are captured on the plate using an anti-nitrotyrosine antibody and the subset of captured proteins that contain histones are then detected using a polyclonal anti-histone antibody. The method detects any analyte that contains both histones and nitrotyrosine. Since histones in serum occur primarily in the form of nucleosomes we refer to this as a NN ELISA rather than nitrated histone ELISA. Nucleosomes nitrated on proteins other than histones would also be detected. Hence, the ELISA detects the presence of nitrated tyrosine residues upon a protein either complexed with histones or upon core histones themselves.

We carried out measurements of NN levels in 397 samples taken from 49 patients at different time points, including times of disease flare and remission. We carried out univariable and multivariable analyses to determine the demographic and clinical factors that are associated with NN levels. We also measured levels of nitrated albumin (NA) as a surrogate marker of overall nitrative stress (given that albumin is a ubiquitous protein in serum) and the levels of anti-nucleosome antibodies (anti-nuc). Anti-nuc levels were measured to test whether they correlate with NN levels, which could be the case if nitration of nucleosomes is important in making them more antigenic.

## Methods

### Patients and samples

Longitudinal serum samples (*n* = 397) were selected retrospectively from a cohort of 49 patients with SLE followed at University College London Hospital (UCLH) with a mean of eight samples per patient (SD 2.16; min 3; max 14) and a mean follow-up of 89 months (SD 46; min 14; max 180). All patients fulfilled the revised American College of Rheumatology (ACR) classification criteria for SLE [10]. We particularly selected patients who had suffered flares of disease activity. Samples were also obtained from 37 healthy control subjects and 38 autoimmune disease controls (13 with rheumatoid arthritis, 12 with myositis and 13 with Sjogren’s syndrome).

For all SLE patient samples where data were available (94% of samples), we obtained anti-dsDNA antibody and complement C3 levels and disease activity from the date of the sample and from the previous three assessments. Anti-dsDNA antibody and C3 levels were measured in the routine clinical laboratory at UCLH using enzyme-linked immunosorbent assay (ELISA) (Shield Diagnostics, Dundee, UK) and laser nephelometry, respectively. Based on the normal limits for our laboratory, anti-dsDNA level >50 IU/ml was defined as high and C3 level <0.9 g/l as low.

Disease activity was measured using the classic British Isles Lupus Assessment Group (BILAG) index [11]. The more recent BILAG 2004 index was not used as many of the samples had been taken before 2004. Current activity (on the date of the sample) for each system was defined as high if the BILAG score was A or B and low if it was C, D or E. Disease activity over the most recent four assessments was characterized as persistently low activity (if BILAG C, D or E was recorded in all systems on each occasion) or persistently moderate-high activity (in the presence of A or ≥1 B score in any BILAG system on at least two out of four occasions). Over 90% of all samples fell into one of those two categories and the rest were excluded from this part of the analysis.

Data on ethnicity, gender, drug therapy and anti-Sm, anti-RNP, anti-Ro and anti-La (all tested by ELISA) antibody status of the patients were obtained from the clinical records of the patients.

Ethical approval was granted by the joint UCL/UCLH Research Ethics Committee and subjects gave informed consent for use of their stored serum samples.

### Serum assays

#### Capture ELISA to detect nitrated nucleosomes

The whole assay was done at room temperature (RT) except where specified and plates were washed three to four times with PBS-0.1% Tween (PBST) between steps. We divided 96-well streptavidin plates in half: the test side was coated with biotinylated polyclonal goat anti-nitrotyrosine antibody (Abcam 27646, VWR Lutterworth, UK) diluted 1:1,000 in PBS and the control side coated with PBS (75 μL per well). After one hour incubation, plates were washed and blocked with 200 μL of 0.5% ovalbumin in PBST (OVA-BST) for one hour. After washing, serum samples were loaded in duplicate onto the plates (100 μL/well) at 1:30 dilution in PBS such that each sample was loaded in two wells on the test side and two matching wells on the control side. After one hour incubation at 37°C and washing, 50 μL per well of rabbit anti-histone H3 antibody (sc-10809, Santa Cruz Biotechnology, Santa Cruz, CA, USA) diluted at 1:2,000 in OVA-BST were added and the plates were incubated for one hour. After washing, 50 μL per well of goat anti-rabbit IgG horse radish peroxidase (HRP) conjugate (Dako P0448, Dako, Ely, UK) diluted at 1:2,000 in 0.5% OVA-BST was added. After incubating for one hour and washing, HRP substrate was added (100 μL per well) and incubated for 10 minutes. The reaction was stopped with 100 μL sulphuric acid and optical density (OD) read at 450 nm. The net OD reading for each sample was calculated by subtracting the OD in the control well from that in the matching test well to exclude non-specific background binding.

In order to be able to compare OD values obtained from different plates on different days, we prepared an in-house standard positive control sample that was loaded in serial dilutions (range 1:15 to 1:120) on every plate. This in-house standard was prepared by pooling serum samples from several patients who had been found to have high serum NN levels in this assay. The mean net OD from duplicate test samples was converted to absorbance units (AU) by comparison to the standard curve of OD for the serial dilutions of the positive control sample on each Plate. A total of 100 AU was defined as the OD given by a 1:30 dilution of the positive control sample. The OD for this 1:30 dilution was reproducibly high, ranging between 1.03 and 1.37.

The NN assay was reproducible with an intra and inter-plate coefficient of variation of <10%.

#### Capture ELISA to detect nitrated albumin

This ELISA protocol was identical to the NN capture ELISA with the following exceptions. The blocking agent was 0.5% agarose in PBS and the anti-histone H3 antibody used was replaced with a rabbit polyclonal anti-human albumin antibody (Abcam 2406).

#### Direct ELISA to detect anti-nucleosome antibodies (IgG)

All steps were carried out at 37°C. The test side of the plate was coated with 50 μL per well of nucleosome antigen (Arotec ATN02-02, Binding Site Ltd, Birmingham, UK) diluted 1:500 in 20 mM Tris/HCL buffer (pH 8.0) containing 0.15 M of NaCl; the control side was coated with buffer alone. Incubation for two hours, then blocking with 1% bovine serum albumin in PBST (BSA-PBST) for one hour were followed by sample loading in duplicate into test and control wells at 1:50 dilution in BSA-PBST. As the positive control, a pooled serum sample was serially diluted (range 1:15 to 1:120) and loaded in the same way. After a 30-minute incubation, goat polyclonal anti-human IgG HRP conjugate (A6029, Sigma, Dorset, UK) diluted at 1:1,000 in BSA-PBST was added. After incubating for 30 minutes, HRP substrate was added and subsequent steps were as for the NN ELISA.

### Statistical methods

The three outcomes in the analyses were serum NN levels, NA levels and anti-nuc levels. All outcomes were found to have a highly positively skewed distribution, which could not be transformed to a more normally distributed scale. Thus, the outcomes were assumed to follow a negative binomial distribution. Due to the longitudinal nature of the cohort, multiple samples for each patient were considered. To allow for the non-independence of the data, multilevel statistical methods were used for analysis. Two level models were used with individual measurements clustered within patients. The analyses, performed using multilevel negative binomial regression, were performed in two stages. First, the separate effect of each factor upon the outcome was examined in a series of univariable analyses. Subsequently, the joint effect of factors was examined in a multivariable analysis. A backward selection procedure was employed to retain only the statistically significant variables. Variance inflation factors were used to assess collinearity between variables and, as a result, some variables that were collinear with other variables were excluded from the multivariable stage of the analysis.

Additional analyses grouped patients as either NN positive or negative depending on their nitrated nucleosome values. Variables measured at the patient level were compared between groups using either Fisher’s exact test for the categorical variables or the unpaired t-test for continuous variables.

Sample level variables were analyzed using multilevel regression methods to allow for the repeat measurements from each patient. Multilevel logistic regression was used to compare binary variables between groups, while multilevel linear regression was used for continuous variables. Continuous variables found to have a positively skewed distribution were given a log transformation before the analysis.

## Results

### Characteristics of subjects

The mean age of the patients with SLE at the time of the earliest sample assayed was 36 years (SD 13.0) and 81% were female. A total of 23 were Caucasian, 18 Afro-Caribbean and 8 other ethnicities. For the healthy controls, the mean age was 31.6 years (SD 6.0) and 62% were female. A total of 27 were Caucasian, 1 Afro-Caribbean and 6 other ethnicities.

Of the 49 patients with SLE, 21 were anti-Ro positive, 6 anti-La positive, 15 anti-RNP positive and 11 anti-Sm positive. During the follow-up period, 29 patients had at least one elevated anti-dsDNA, 32 had at least one low C3 and 46 suffered at least one flare (BILAG A or B in at least one system). Flares in all eight systems of the classic BILAG index were represented in the cohort.

### Serum NN levels are higher in patients with SLE than in healthy controls or patients with other autoimmune diseases

**Figure 1:**
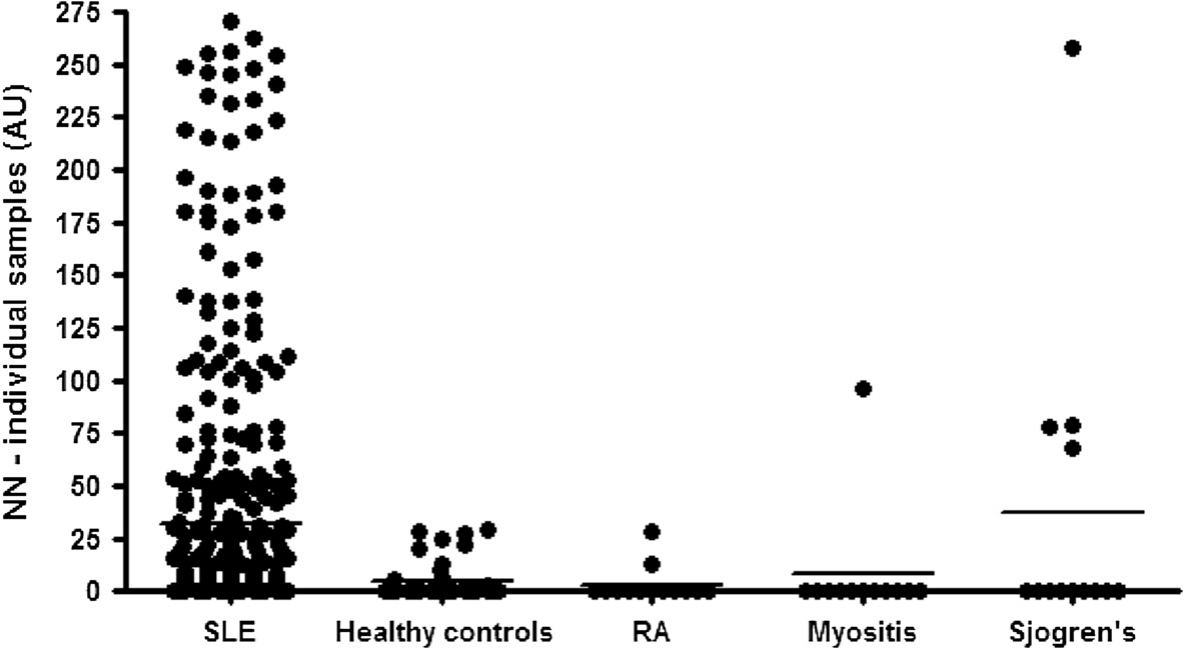
**NN levels in patients with SLE compared to healthy and autoimmune disease controls.** This figure shows the results obtained in the NN ELISA on testing serum samples from patients with SLE in comparison with healthy controls and patients with other autoimmune rheumatic diseases (myositis, rheumatoid arthritis and Sjogren’s syndrome). NN levels were higher in the SLE group than the other groups (*P* = 0.01 by one way ANOVA/Kruskal Wallis test). NN, nitrated nucleosomes; SLE, systemic lupus erythematosus.

Figure 1 shows that mean levels of serum NN in patients with SLE were significantly higher in patients with SLE than in healthy controls or patients with other autoimmune diseases - rheumatoid arthritis, myositis or Sjogren’s syndrome (*P* = 0.01 by one-way ANOVA/Kruskal Wallis test). We do not have formal disease activity measurements for the patients with these other diseases but in most cases, samples were taken when patients were symptomatic. Since NN levels were very low in 31/38 of these patients, we do not believe that active rheumatoid arthritis, myositis or Sjogren’s syndrome is associated with raised NN levels.

### Serum NN are present in 63% of patients with SLE and the levels of NN in these patients vary over time

Of the 49 patients with SLE tested, 18 never had detectable serum NN, whereas the other 31 (63%) had serum NN levels that varied significantly over time (mean 32 AU, SD 62.2, range 0 to 270). The characteristics of the 18 NN-negative patients and the other 31 patients are compared in Table 1. All patients who never had NN were anti-Sm negative, whereas 35% of the others were anti-Sm positive (*P* = 0.004). This clear dichotomy did not apply to any of the other antibody specificities that were tested, though the mean anti-dsDNA level in NN-positive patients was almost twice that seen in NN-negative patients. A total of 63.2% of NN-positive samples, but only 25.2% of NN-negative samples, came from patients who were taking immunosuppressants (*P* = 0.001).

**Table 1:** Comparison of patients with SLE who never had serum NN with the rest of the group

	**NN-negative**	**NN-positive**	***P*-value**
	**(n patients = 18)**	**(n patients = 31)**	
	**(n samples = 144)**	**(n samples = 253)**	
Mean age at first sample (SD)^(*)^	37.7 (13.1)	34.9 (12.8)	0.47
% Female (n)^(*)^	77.8 (14)	83.9 (26)	0.71
Ethnicity^(*)^			0.41
% AC (n)	27.8 (5)	41.9 (13)	
% C (n)	61.1 (11)	38.7 (12)	
% Other (n)	11.1 (2)	19.3 (6)	
Median anti-dsDNA IU/ml (IQR)^(**) (†)^	29 (13, 97)	82 (17, 171)	0.18
Mean C3 g/l (SD)^(**)^	1.0 (0.2)	0.9 (0.3)	0.10
Median Global BILAG score (IQR)^(**) (†)^	5 (2, 8)	5 (3, 10)	0.22
% anti-Sm positive (n)^(*)^	0.0 (0)	35.5 (11)	**0.004**
% anti-RNP positive (n)^(*)^	27.8 (5)	32.3 (10)	1.00
% anti-Ro-positive (n)^(*)^	38.9 (7)	45.2 (14)	0.77
% anti-La positive (n)^(*)^	16.7 (3)	9.7 (3)	0.66
% on hydroxychloroquine (n)^(**)^	42.1 (61)	44.7 (113)	0.66
% on immunosuppressants (n)^(**)^	25.7 (37)	63.2 (160)	**0.001**
% on ≥5 mg/day corticosteroids (n)^(**)^	68.1 (98)	78.3 (198)	0.31

^(*)^Analysis performed at the patient level.

^(**)^Analysis performed at the sample level.

^(†)^Statistical comparison performed on log scale.

Footnote - Global BILAG score is calculated from the formula A = 9, B = 3, C = 1, D = 0, E = 0 as described in previous papers [12]. Bold type indicates statistically significant *P*-values (*P* <0.05). BILAG, British Isles Lupus Assessment Group; NN, nitrated nucleosomes; RNP, ribonucleoprotein; SLE, systemic lupus erythematosus.

**Figure 2:**
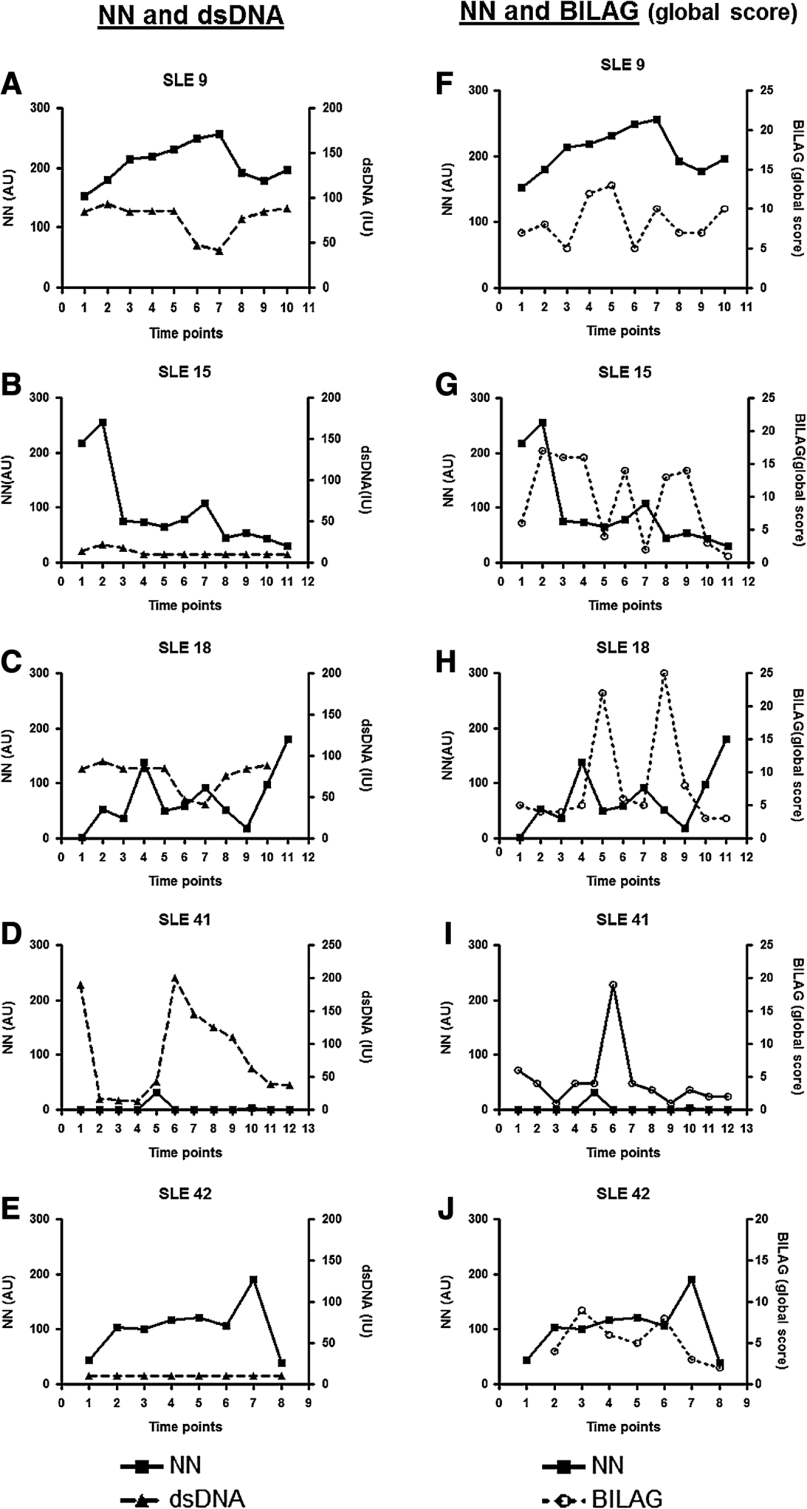
**Variation of NN and anti-dsDNA levels and global BILAG scores over time.** This figure shows the variation of serum NN levels over time in five patients compared to anti-dsDNA levels **(A-E)** and global BILAG scores **(F-J)**. anti-dsDNA, anti-double stranded DNA antibodies; BILAG, British Isles Lupus Assessment Group; NN, nitrated nucleosomes.

Figure 2 shows the variation of serum NN levels over time in five patients with SLE (patients SLE 9, 15, 18, 41 and 42) compared to variations in anti-dsDNA level (Figures 2A-E) and global BILAG score (Figures 2F-J). None of these patients showed close relationships between anti-dsDNA and NN over time. In some cases, such as SLE 9, 15 18 and 42, NN levels follow disease activity more closely than anti-dsDNA. This is especially striking in cases SLE 15 and 42, where anti-dsDNA is always low. However, there are other cases where the NN level is persistently low and anti-dsDNA follows activity more closely (patient SLE 41) and it is important to remember that approximately a third of patients never had raised NN levels.

### Clinical, demographic and serological variables associated with serum NN and NA levels in patients with SLE

Our hypothesis was that the levels of NN in patients with SLE would be affected both by the overall level of nitration and by the level of serum nucleosomes present as a target for nitration. If this were true, there should be detectable differences between the variables influencing serum NN levels and those influencing NA levels. These differences clearly exist, as shown in Table 2, which includes results from univariable analysis of 24 factors.

**Table 2:** Univariable analysis of factors associated with serum NN and NA levels in patients with SLE

**Variable**	**Category/term**	**Ratio for NN (95% CI)**	***P*-value for NN**	**Ratio for NA (95% CI)**	***P*-value for NA**
Gender *	Female (n = 336)	1		1	
	Male (n = 61)	2.01 (1.06, 3.81)	**0.03**	5.00 (2.50,9.98)	**<0.001**
Disease duration^†^	Ratio given per 5-year increase	0.43 (0.23, 0.79)	**0.005**	0.32 (0.20,0.51)	**<0.001**
Age (at diagnosis)^†^	Ratio given per 10-year increase	0.10 (0.02, 0.46)	**<0.001**	0.04 (0.01,0.16)	**0.002**
Ethnicity*	Caucasian (n = 182 )	1		1	
	Afro-Caribbean (n = 146 )	0.75 (0.47, 1.22)		2.07 (1.26,3.40)	
	Other (n = 69)	0.33 (0.17, 0.63)	**0.003**	3.75 (1.86,7.56)	**<0.001**
Any ENA**	No (n = 165)	1		1	
	Yes (n = 232)	3.43 (2.19, 5.37)	**<0.001**	3.19 (1.93,5.27)	**<0.001**
Anti-Ro**	No (n = 226)	1		1	
	Yes (n = 171)	1.70 (1.12, 2.59)	**0.01**	1.81 (1.14,2.89)	**0.01**
Anti-La**	No (n = 345)	1		1	
	Yes (n = 52)	0.79 (0.41, 1.50)	0.47	0.46 (0.20,1.06)	0.07
Anti-Sm**	No (n = 298)	1		1	
	Yes (n = 99)	5.63 (3.66, 8.67)	**<0.001**	6.25 (3.84,10.2)	**<0.001**
Anti-RNP**	No (n = 288)	1		1	
	Yes (n = 109)	1.08 (0.69, 1.70)	0.74	1.72 (1.06,2.79)	**0.03**
Anti-dsDNA level	<50 IU/ml (n = 177)	1		1	
	≥50 IU/ml (n = 170)	0.76 (0.26, 1.13)	0.17	1.32 (0.66,2.65)	0.43
C3 level	<0.9 g/l (n = 139)	1		1	
	≥0.9 g/l (n = 208)	0.83 (0.57, 1.20)	0.32	1.26 (0.86,1.87)	0.24
Disease activity in general system§	A, B (n = 31)	1		1	
	C, D, E (n = 344)	0.96 (0.60, 1.54)	0.87	1.19 (0.71,2.00)	0.52
Disease activity in mucocutaneous system	A, B (n = 41)	1		1	
	C, D, E (n = 334)	0.99 (0.63, 1.56)	0.95	1.32 (0.76,2.29)	0.33
Disease activity in neuropsychiatric system	A, B (n = 18)	1		1	
	C, D, E (n = 357)	0.56 (0.13, 2.38)	0.44	1.04 (0.50,2.19)	0.91
Disease activity in musculoskeletal system	A, B (n = 47)	1		1	
	C, D, E (n = 328)	1.20 (0.47, 3.04)	0.71	0.68 (0.43,1.05)	0.08
Disease activity in cardiorespiratory system	A, B (n = 13)	1		1	
	C, D, E (n = 362)	0.43 (0.24, 0.78)	**0.006**	1.40 (0.22,8.70)	0.72
Disease activity in vascular system	A, B (n = 12)	1		1	
	C, D, E (n = 363)	0.33 (0.17, 0.63)	**0.001**	0.39 (0.19,0.82)	**0.01**
Disease activity in renal system	A, B (n = 41)	1		1	
	C, D, E (n = 329)	1.09 (0.40, 2.97)	0.86	4.28 (2.13,8.58)	**<0.001**
Disease activity in hematological system	A, B (n = 95)	1		1	
	C, D, E (n = 280)	1.21 (0.84, 1.74)	0.30	1.44 (0.95,2.17)	0.08
Overall disease activity over last four assessments	Persistently low (n = 166)	1		1	
	Persistently mod/high (n = 209)	0.91 (0.66, 1.25)	0.57	0.66 (0.47,0.92)	**0.01**
Hydroxychloroquine	No (n = 223)	1		1	
	Yes (n = 174)	3.52 (2.40, 5.17)	**<0.001**	3.21 (2.08,4.95)	**<0.001**
Immunosuppression	No (n = 200)	1		1	
	Yes (n = 197)	1.66 (1.17, 2.34)	**0.004**	1.33 (0.89,1.98)	0.16
Oral corticosteroids	≤7.5 mg/day (n = 101)	1		1	
	>7.5 mg/day (n = 296)	0.81 (0.59, 1.13)	0.22	1.00 (0.69,1.44)	0.99
Albumin	Ratio given per 5 g/l increase	1.38 (1.20,1.59)	**<0.001**	1.42 (1.22,1.65)	**<0.001**

*For gender, n values refer to the numbers of samples taken from female and male subjects, rather than the numbers of females and males in the cohort of patients. A similar stipulation applies to ethnicity where n values refer to the number of samples taken from patients of each ethnic group.

^†^For the complex associations with disease duration and age, we carried out analysis of both linear and squared terms. The figures in the table refer to linear terms. The ratios for squared terms for NN are 0.66 (95% CI: 0.51, 0.85) for disease duration and 1.56 (95% CI: 1.20, 2.03) for age. The ratios for squared terms for NA are 0.59 (95% CI: 0.46, 0.75) for disease duration and 1.72 (95% CI: 1.32, 2.24) for age.

**For ENA, anti-Ro, anti-La and anti-Sm we did not have results from the date of every sample but it is assumed that positivity and negativity for these antigens generally remain stable.

§“Disease activity in general system” refers to the BILAG score (A, B, C, D or E) in the General Category of the BILAG index on the day when each sample was taken. The same principle applies to the remaining organ systems listed in the table, which are the eight different categories recorded in BILAG. Bold type indicates statistically significant *P*-values (*P* <0.05).

NN levels were twice as high in men as in women. NA levels were five times as high in men as in women. The effects of ethnicity on NA are the opposite of the effects on NN. Caucasians had the lowest NA levels but highest NN levels of any ethnic group. For both NN and NA there was a complex non-linear relationship with both disease duration and age at diagnosis. NN levels were lowest in very young and very old subjects with a peak NN level at age 30 and at disease duration of eight years.

Anti-Sm positivity was strongly associated with both elevated NN and elevated NA.

We did not detect any association with the commonly used serological markers of active lupus (elevated anti-dsDNA and low C3) or with persistent overall disease activity for either NN or NA. Considering the individual systems of the body, we found that vasculitis flares (that is, A or B score in the vasculitis domain of BILAG) were associated with significantly raised serum NN and NA. NN but not NA was raised in cardiorespiratory flares. Renal flares were associated with a significant decrease in NA but not in NN. There were no statistically significant differences between flare (A/B) and non-flare (C/D/E) samples for other systems but for neuropsychiatric flares the NN levels in C/D/E samples were approximately 50% of those in A/B samples. This difference did not reach statistical significance due to wide confidence intervals. There was no effect of neuropsychiatric flares on NA levels.

Treatment with hydroxychloroquine was associated with elevated NN and NA levels. Immunosuppressants (including mycophenolate, azathioprine, methotrexate and cyclophosphamide) were only associated with elevated NN, whereas high dose corticosteroid treatment was associated with neither NN nor NA.

Tables 3 and 4 show the results of multivariable analysis of associations with NN and NA, respectively. Disease duration, ethnicity, negative anti-La, albumin and low renal disease activity are all associated with NA but not NN, whereas hydroxychloroquine and high vasculitis disease activity are associated with NN but not NA. Age at diagnosis, anti-Sm antibody positivity and treatment with immunosuppressants all show similar associations with both NN and NA levels. In particular, anti-Sm positivity shows a very strong independent association with both NN and NA (*P* <0.001 for both).

**Table 3:** Multivariable analysis of factors associated with serum NN levels in patients with SLE

**Variable**	**Category/term**	**Ratio (95% CI)**	***P*-value**
Age (at diagnosis) *	Age	0.02 (0.003, 0.11)	**<0.001**
	Age	2.12 (1.64, 2.76)	
Anti-Sm	No	1	
	Yes	6.31 (3.39, 11.7)	**<0.001**
Disease activity in vascular system	A, B	1	
	C, D, E	0.40 (0.24, 0.69)	**0.001**
Hydroxychloroquine	No	1	
	Yes	1.96 (1.09, 3.53)	**0.02**
Immunosuppression	No	1	
	Yes	2.96 (1.97, 4.46)	**<0.001**

*Ratio given for 10-year increase. NN, nitrated nucleosomes; SLE, systemic lupus erythematosus.

**Table 4:** Multivariable analysis of factors associated with serum NA levels in patients with SLE

**Variable**	**Category/term**	**Ratio (95% CI)**	***P*-value**
Disease duration	Ratio given per five-year increase	2.24 (1.55, 3.26)	**<0.001**
Age (at diagnosis) *	Age	0.01 (0.00, 0.04)	**<0.001**
	Age^2^	2.58 (1.86, 3.56)	
Ethnicity	Caucasian	1	
	Afro-Caribbean	2.99 (1.35,6.65)	
	Other	14.9 (5.82, 38.0)	**<0.001**
Anti-La	No	1	
	Yes	0.16 (0.06, 0.49)	**<0.001**
Anti-Sm	No	1	
	Yes	28.2 (14.4, 55.3)	**<0.001**
Disease activity in renal system	A, B	1	
	C, D, E	3.78 (2.07,6.90)	**<0.001**
Immunosuppression	No	1	
	Yes	1.45 (1.02, 2.05)	**0.04**
Albumin	Ratio per 5 g/l increase	1.12 (1.01,1.24)	**0.03**

*Ratio given for a 10-year increase. NA, nitrated albumin; SLE, systemic lupus erythematosus.

### There is no association between serum levels of NN and anti-nuc in multivariable analysis and different variables are associated with these outcomes

Both univariable and multivariable analyses of the factors associated with serum anti-nuc levels were performed. Only the results of multivariable analysis are presented here (in Table 5), as use of anti-nuc as a biomarker in SLE has been studied extensively by other authors [13-15] and is not the subject of our paper.

**Table 5:** Multivariable analysis of factors associated with serum anti-nuc levels in patients with SLE

**Variable**	**Category/term**	**Ratio (95% CI)**	***P*-value**
Gender	Female	1	
	Male	2.83 (1.72, 4.69)	**<0.001**
Disease duration	Ratio given per 5-year increase	0.87 (0.78, 0.98)	**0.02**
Age (at diagnosis)	Ratio given per 10-year increase	0.59 (0.49, 0.72)	**<0.001**
RNP	No	1	
	Yes	0.47 (0.32, 0.70)	**<0.001**
dsDNA	Ratio given per 10-fold increase (1 unit on log scale)	1.79 (1.40, 2.28)	**<0.001**
C3	Ratio given per 0.1 g/l increase	0.29 (0.15, 0.56)	**<0.001**
NN level	Ratio given per 10-fold increase (1 unit on log scale)	0.88 (0.72,1.07)	**0.20**

Anti-nuc, Anti-nucleosome antibody; dsDNA, double stranded DNA; RNP, ribonucleoprotein; SLE, systemic lupus erythematosus.

Samples obtained from men had higher anti-nuc levels than samples from women. Both increasing age at diagnosis and increasing disease duration were associated with significant reductions in anti-nuc levels. Increasing anti-nuc levels were associated with elevated anti-dsDNA as noted by previous authors [14,15] and with low C3. In contrast to the results obtained with NN and NA, there was no relationship between anti-nuc and anti-Sm status. There were no associations between treatment with hydroxychloroquine or immunosuppressants and levels of anti-nuc.

Most importantly from the point of view of the present study, there was no significant association between NN and anti-nuc levels on multivariable analysis suggesting that nitration probably does not affect the immunogenicity of nucleosomes.

## Discussion

In this paper we describe a novel capture ELISA that measures serum levels of NN. Mean NN levels are significantly higher in patients with SLE than in healthy controls or in patients with other autoimmune rheumatic diseases but there is a subset of patients with SLE who never test positive for NN. These persistently NN-negative patients comprise about one-third of the total population and have lower disease activity and anti-dsDNA antibody levels and less use of immunosuppressants than the other two-thirds. NN-negative patients are all anti-Sm antibody negative.

Currently, available serum biomarkers for monitoring patients with SLE are antibodies (particularly anti-dsDNA) or markers of immune activation, such as depleted complement levels. Potential new serum biomarkers include pro-inflammatory chemokines [16]. The ELISA described in our current paper is novel in that, rather than an antibody or cytokine, it measures levels of an antigen, which has been chemically modified *in vivo*. The critical importance of nucleosomes in the pathogenesis of lupus nephritis has been established by a number of authors. Berden and colleagues showed that nucleosome/anti-nucleosome complexes can cause nephritis in murine models of SLE [9,17]. The elegant electron microscopy studies of Rekvig’s group demonstrated that deposited IgG co-localizes with electron-dense chromatin structures in renal biopsies from human and murine lupus nephritis [18,19]. More recently, Kanapathipillai *et al.* showed a direct stimulatory effect of nucleosomes alone (not requiring complexed antibodies) on expression of chemokines by mesangial cells from NZB/W F1 mice [20]. There is also evidence that chemical modification of nucleosomes, specifically hyperacetylation, can alter their biological effects in SLE. Dieker *et al.* demonstrated that samples from 26 of 35 patients with SLE bound more strongly to a triacetylated 22-amino acid peptide from histone H4 than to the non-acetylated version of the same peptide [21]. Subcutaneous administration of the triacetylated, but not the non-acetylated, peptide accelerated development of proteinuria and increased mortality in MRL/*lpr* mice [21] and this effect was not mediated via an increase in anti-nucleosome antibody levels. In the same paper, this group showed that hyperacetylated nucleosomes stimulated expression of co-stimulatory molecules and pro-inflammatory cytokines by dendritic cells (DC) from MRL/*lpr* mice [21]. However, they did not measure levels of hyperacetylated nucleosomes in the serum of humans or mice. Our current paper is the first to look specifically at nitration of nucleosomes and the first to measure serum levels of modified nucleosomes in patients with SLE. We found that elevated NN level is itself a specific marker for SLE and is also strongly associated with another SLE-specific biomarker in anti-Sm. Though these are promising findings, limitations of our work include the absence of strong association with overall disease activity and expression of results in terms of binding of our in-house control serum sample. If NN is to be a useful biomarker in patients with SLE, a more generally applicable standard must be found. In a recently published paper, Pratesi *et al.*[22] showed that sera from patients with rheumatoid arthritis, but not from healthy controls or patients with other autoimmune rheumatic diseases, contained antibodies to deiminated histone H4. They postulated that the deiminated H4 autoantigens could derive from neutrophil extracellular traps in these patients. A modification of our NN assay, using anti-citrulline rather than anti-nitrotyrosine as the capture antibody, could potentially be used to detect deiminated histones in the serum of patients with rheumatoid arthritis.

There was a strong association between anti-Sm positivity and nitration in the multivariable analysis of factors associated with NN and NA levels. To our knowledge this association between nitrative stress and anti-Sm positivity has not been described before. The Sm antigen is a complex of ribonucleoproteins in the spliceosome [23]. Anti-Sm antibodies were first recognized in 1966 [24], and are highly specific to patients with SLE. Anti-Sm antibodies have been found in lupus nephritis renal biopsies [25]. Some retrospective studies reported associations between anti-Sm positivity and psychosis [26] or nephritis [27], while others found no association between anti-Sm antibodies and any clinical outcome [28]. A large Chinese study compared 469 anti-Sm antibody positive and 1,115 anti-Sm antibody negative patients with SLE [29]. The anti-Sm positive patients had higher disease activity, higher anti-dsDNA antibody and lower C3 levels than anti-Sm negative patients. Vasculitis occurred more often in anti-Sm positive than in anti-Sm negative patients (13.7% vs 7.4%, *P* <0.05). Our current results show strong associations among NN levels, anti-Sm and vasculitis.

Increased nitration may promote development of anti-Sm antibodies. Experiments on mice transgenic for the rearranged heavy chain variable region genes of a monoclonal anti-Sm antibody [30] showed that immature DC carrying surface Sm antigen interacted directly with B cells leading to the production of anti-Sm antibody. These DC obtained Sm antigen partly from apoptotic cell debris, which also contains nucleosomes complexed to the High Mobility Group Box 1 (HMGB1) protein [31]. HMGB1 can activate DC [32] and also promotes NO production by phagocytes. Thus nucleosome/HMGB1 complexes could stimulate the DC-B cell interaction that leads to anti-Sm antibody production as well as driving NO production. It is not known whether nitration of HMGB1 itself alters its pro-inflammatory properties. However, a recent study [33] has shown that altering the redox state of HMGB1 through the oxidation of free thiol cysteines to form disulfide bonds makes it more pro-inflammatory. If nitration of nucleosome/HMGB1 complexes likewise promotes their pro-inflammatory properties this would create a potential positive feedback loop, and this is a mechanism that warrants further exploration. It may also be interesting to look at nitration of the Sm antigen.

NA and NN levels were associated with use of immunosuppressants. This association is unlikely to arise simply from the fact that immunosuppressants are used more commonly in highly active disease, because persistent clinical disease activity, anti-dsDNA antibody and C3 levels were not associated with either NA or NN levels. In future studies it may also be interesting to look at correlations with erythrocyte sedimentation rate and/or C-reactive protein but we do not have data on those variables for this study. We collected data on anti-dsDNA and C3 because those biomarkers are more closely associated with flares in SLE. The drugs themselves could promote nitration, but we know of no evidence supporting this. Cyclosporin can promote tyrosine nitration in endothelial cells [34], but only two samples in this study were from patients taking cyclosporin and both were negative for NN.

A large inception cohort study has shown that 40% of 1,206 patients with SLE suffered from neuropsychiatric symptoms, which were associated with reduced quality of life but that in the majority of cases these were not due to active inflammation and did not require immunosuppression [35]. It is important, however, to identify the subpopulation of patients in whom these symptoms are due to active cerebral SLE. With appropriate treatment, the symptoms are significantly more likely to resolve in those patients compared to patients in whom the symptoms are not due to active SLE [35]. Current imaging techniques and blood tests do not accurately distinguish patients with active neuropsychiatric SLE from those who have neuropsychiatric symptoms not caused by SLE. Our results suggesting that elevated NN levels could potentially be a marker for neuropsychiatric flares are based on results from 11 different patients taken over a 12-year period. Over 70% of the neuropsychiatric manifestations in these patients were headaches and seizures. It is important to extend these results by testing larger numbers of samples from patients with a wider range of neuropsychiatric manifestations.

Vasculitis is a relatively uncommon manifestation of SLE [29]. In multivariable analysis we found a statistically significant association of high NN (but not NA) with vasculitis based on results from nine different patients over an 11-year period. Review of the medical records showed that all these patients had cutaneous vasculitis at the time of their flares. It is possible that raised NN levels in patients without visible vasculitis may be a marker of subclinical vascular activation. It will, therefore, be interesting to see whether NN levels are associated with objective measures of atherosclerosis, such as carotid ultrasound [2]. This potential association is underlined by the fact that peroxynitrite, a powerful nitrating and oxidizing agent, is generated in atherosclerotic plaques. If such an association exists, then NN levels may be relevant to assessment of CVD risk in patients with SLE.

## Conclusion

By developing a novel assay to measure serum NN levels we have demonstrated that these levels are raised in patients with SLE compared to healthy controls and patients with other autoimmune rheumatic diseases. NN-positivity is strongly linked to anti-Sm antibody positivity and may be a marker for neuropsychiatric flares and vasculitis in patients with SLE. Further studies in larger numbers of patients with these manifestations are required.

## Abbreviations

Anti-dsDNA: Anti-double stranded DNA antibodies; Anti-nuc: Anti-nucleosome antibody; AU: Absorbance units; BILAG: British Isles Lupus Assessment Group index (for measuring disease activity in SLE); BSA-PBST: Bovine serum albumin in PBST; CVD: Cardiovascular disease; DC: Dendritic cells; ELISA: Enzyme-linked immunosorbent assay; HMGB1: High Mobility Group Box 1 protein; HRP: horse radish peroxidase; NA: Nitrated albumin; NN: Nitrated nucleosomes; NO: Nitric oxide; OD: Optical density; OVA-BST: Ovalbumin in PBST; PBS: Phosphate-buffered saline; PBST: PBS-0.1% Tween; SLE: Systemic lupus erythematosus; UCLH: University College London Hospital.

## Competing interests

The authors have no competing interests.

## Authors’ contributions

YI and AR conceived the idea of the project. YI, CP and KFA developed and optimized the novel nitrated nucleosome assay. SC carried out the experimental work. PB and SC carried out statistical analysis. IG, DI, YI, AR, SC and DL took part in the design of the study and analysis of data. SC and AR wrote the final paper. All authors revised the paper and agreed to the final version.
